# Integrating newborn screening for spinal muscular atrophy into health care systems: an Australian pilot programme

**DOI:** 10.1111/dmcn.15117

**Published:** 2021-11-28

**Authors:** Arlene M D'Silva, Didu S T Kariyawasam, Stephanie Best, Veronica Wiley, Michelle A Farrar, NSW SMA NBS Study Group, Anja Ravine, David Mowat, Hugo Sampaio, Ian E Alexander, Jacqui Russell, Kristi Jones, Zena Junek

**Affiliations:** ^1^ Department of Neurology Sydney Children’s Hospital Network Sydney New South Wales Australia; ^2^ School of Women’s and Children’s Health University of New South Wales Medicine and Health University of New South Wales Sydney New South Wales Australia; ^3^ Australian Institute of Health Innovation Macquarie University Sydney New South Wales Australia; ^4^ Australian Genomics Murdoch Children’s Research Institute Melbourne Victoria Australia; ^5^ NSW Newborn Screening Programme Children’s Hospital Westmead Westmead New South Wales Australia; ^6^ Discipline of Child and Adolescent Health Sydney Medical School Faculty of Medicine and Health University of Sydney Sydney New South Wales Australia

## Abstract

**Aim:**

This study dynamically designed, evaluated, and implemented the components of an Australian newborn bloodspot screening (NBS) pilot programme for spinal muscular atrophy (SMA).

**Method:**

We used an implementation‐effectiveness study design and continuous interdisciplinary review to measure SMA NBS test protocol performance, identify and overcome laboratory and clinical barriers to implementation, and describe progress during the 2‐year pilot study.

**Results:**

The NBS programme screened 252 081 newborn infants from 1st August 2018 to 31st January 2021. Using an NBS pilot test protocol, 21 infants were diagnostically confirmed with SMA. The NBS pilot test protocol had a sensitivity of 100%, specificity greater than 99.9%, false‐positive rate less than 0.001%, a false‐negative rate of 0%, and positive predictive value of 95.5%. A severe phenotype was predicted on the basis of two copies of *SMN2* in 57.2% of newborn infants screening positive for SMA. Clinical signs consistent with SMA were evident in 6 out of 21 screen‐positive newborn infants within the first 4 weeks of life. A multidisciplinary team establishing strong partnerships across clinical and laboratory staff was key to implementation.

**Interpretation:**

This pilot programme suggests that NBS is essential for early identification of newborn infants at risk of SMA and can be effectively translated into clinical practice.

## Abstract 2

This original article is commented by Mueller‐Felber on page 535 of this issue.

**Abbreviations**
CMAP: Compound muscle action potential
NBS: Newborn bloodspot screening
SMA: Spinal muscular atrophy

**What this paper adds**

- Newborn bloodspot screening (NBS) maximized opportunities for early intervention through timely and equitable spinal muscular atrophy diagnosis.
- A multidisciplinary team and strong partnerships among stakeholders are key to implementation of NBS.

Newborn bloodspot screening (NBS) continues to be one of the most successful population health programmes, yielding greatly improved health outcomes for identified cases achieved by a combination of very early diagnosis and expedient initiation of treatment and management.^1^ New technologies and innovative treatments are now improving the capacity to identify many previously untreatable serious disorders, which could be included in NBS programmes. An exemplar is spinal muscular atrophy (SMA), wherein genetic therapies have transformed the clinical landscape from a lethal to a treatable disease. In this condition, a shift towards pre‐ or early symptomatic treatment optimizes outcomes for patients, before irreversible motor neuron loss.^2^ Results from interventional studies in treated presymptomatic infants with fewer than four survival motor neuron 2 (*SMN2*) gene copies have demonstrated maintenance of respiratory and bulbar function and achievement of typical motor developmental milestones.^3^ Accordingly, increasing numbers of NBS programmes are adding SMA to screening panels, using genetic technologies as a first‐tier assay on dried bloodspots to identify deletion in exon 7 of the survival motor neuron 1 (*SMN1*) gene which forms the genetic basis of disease.^4^, ^5^, ^6^, ^7^

Enhancing the quality, efficiency, and effectiveness of NBS services to care for infants and families with SMA is important for improving utility, facilitating sustainability, and maintaining public trust. To support NBS readiness and inclusion of new disorders into NBS programmes, barriers and facilitators have been identified including establishing screening algorithms, follow‐up protocols, and education materials for each condition.^8^ Recommended international best practice for NBS services highlights the importance of seamless integration of laboratory and clinical services for facilitating rapid diagnostic confirmation, early treatment and providing long‐term support for patients and families, as well as effective programme evaluation.^9^

The New South Wales and the Australian Capital Territory NBS pilot programme for SMA started in August 2018 to inform assessment of practice and policy requirements for inclusion of SMA as a routine element within national NBS. Our initial report described the screening and diagnostic pathway (efficiency and timelines), clinical characteristics, and short‐term health outcomes for the first 12 months for our screen‐positive cohort.^10^

In the current study, we describe the development and implementation of a patient‐centred programme that enables earliest possible diagnosis and therapeutic intervention. We outline the challenges encountered and solutions implemented to establish a framework that enables timely delivery of first‐tier screening genomic technologies and innovative therapies that include use of antisense oligonucleotides, gene therapies, and oral splicing modifiers. We envisage that findings from the pilot programme will guide the successful translation of new genomic technologies and NBS services for SMA into clinical practice and sustainable health policy.

## METHOD

### The setting and NBS procedures

Each year the New South Wales and the Australian Capital Territory NBS programme offers screening for over 30 congenital conditions among about 100 000 newborn infants across a total area of 803 508km^2^, detecting approximately 100 newborn infants who require urgent clinical assessment and treatment. Participation in the programme is strongly encouraged, rather than being compulsory. Parents’ attention is drawn to the recently added SMA pilot and that it is an opt‐out programme. Affected newborn infants and their families are supported by the Australian public health system, which provides primary care, public hospitals, specialist services, and outpatient pharmaceuticals.

Details of the development of the SMA‐related screening procedures, including consent, methodology, screening, and diagnostic and post‐screening surveillance pathways, have been described previously.^10^ Infants with screening results indicating absence of *SMN1* exon 7 alleles were classified as screen positive. This methodology did not detect newborn infants with heterozygous deletions or point mutations on the *SMN1* gene (constituting 2%–5% of the population with SMA). To enable proficiency testing during the pilot study, *SMN2* copy number did not determine screen positivity.

### Study design and participants

A hybrid type 2 implementation‐effectiveness study design alongside the SQUIRE guidelines was used, with simultaneous evaluation of outcomes (time frames, coverage, sensitivity, specificity, false‐positive rate, false‐negative rate, positive predictive value for tested dried bloodspots after initial laboratory validation) and implementational factors.^11^, ^12^ The knowledge‐to‐action process framework, a dynamic, iterative process involving engagement among relevant stakeholders (including laboratory scientists, clinicians, and families), was adopted to provide a systematic structure to assess practices, interventions, and health outcomes across four domains. The four domains include knowledge of the condition, screening and diagnostic testing for SMA, SMA clinical care and supporting families as a central focus, and impact of SMA screening on the NBS programme as a whole.^13^

We focused on working collaboratively to identify barriers to implementation across the NBS pathway. Strategies to overcome these barriers were developed by auditing practices and obtaining feedback from stakeholders to enhance health care delivery. Iterative steps for dissemination of findings and practice improvements were shared with stakeholders in real time. We continued to devise and plan next steps for future practice, strategies to determine and evaluate meaningful health outcomes, and to develop pathways to ensure sustained knowledge use.

A centralized statewide paediatric neuromuscular and molecular genetic laboratory service is provided within Sydney Children’s Hospital Network, enabling surveillance for clinically detected cases of SMA. Infants detected by the pilot programme from 1st August 2018 to 31st January 2021 were referred to this service where demographic and clinical characteristics were collated. Clinical assessments were augmented with neurophysiological studies. The maximum amplitude of compound muscle action potentials (CMAPs) of the peroneal nerve (tibialis anterior) or ulnar nerve (abductor digiti minimi) was recorded at diagnosis, with a minimum of three G1 electrode positionings to ensure measurement of maximum amplitude. Longitudinal assessments were undertaken in a subset of infants alongside initiation of disease‐modifying therapy.

### Ethics approval

Ethics approval was gained from the Sydney Children’s Hospital Network Human Research Ethics Committee (HREC LNR/18/SCHN/307). Written informed consent was obtained from participating families.

### Data analysis

Descriptive statistics were used to analyse data and expressed as a number (percentage) and median (range, standard deviation). Throughout statistical analysis, normal quantile–quantile plots of residuals and Shapiro–Wilk tests demonstrated no gross deviations from the normal distribution. For initial CMAP measurements from tibialis anterior, the differences in means between infants with SMA with two and three SMN2 copies were tested with Student’s *t*‐tests. A paired Student’s *t*‐test was used to compare longitudinal CMAPs from abductor digiti minimi within the first 5 weeks of therapy. Implementational challenges and learnings arising from them were captured prospectively from weekly neuromuscular multidisciplinary team meetings, real‐time feedback from families, and ad hoc laboratory discussions. Two reviewers (MF and AD) examined the meeting content, written communications, and interpersonal exchanges applying deductive content analysis to structure shared experiences, learnings, local challenges, and exchange of knowledge using the Australian National Policy Framework for NBS (Table S1, online supporting information).^14^

## RESULTS

### Participants’ characteristics

During the study interval, 252 081 newborn infants were included in the NBS programme. Twenty‐two newborn infants were identified as screen positive for SMA. Diagnostic testing confirmed that 21 of the 22 screen‐positive infants had homozygous deletions in exon 7 of *SMN1*. The atypical screen‐positive result necessitated an individualized genetic and clinical approach (Appendix S1, online supporting information). Among the 21 infants with confirmed SMA, 12 had two *SMN2* copies, eight had three copies, and one had four copies (Table S2, online supporting information). The median time between birth and a screen‐positive result for these cases was 3 days (range 2–15) and the median time between birth and diagnostic confirmation was 15 days (range 10–23d). The median time to the start of therapeutic intervention was 25 days (range 15–39d). A further two infants with SMA were diagnosed clinically. For one of these, routine tandem mass spectrometry was completed on the NBS sample; however, *SMN1* was not analysed owing to system errors that occurred during establishment of the pilot and before integration into laboratory computerized management systems. The laboratory did not receive an NBS sample for the other case. These were not related to the ability of the screening test to identify neonates correctly. Both presented clinically at 4 months of age.

### Implementation knowledge and actions

Our experiences, learnings, and challenges were classified into (1) knowledge of the condition, (2) screening and diagnostic testing for SMA, (3) SMA clinical care and supporting families as a central focus, and (4) the impact of screening for SMA on the NBS programme (Table 1).

**Table 1:** Challenges, learnings, and strategies used during the newborn bloodspot screening (NBS) pilot programme for spinal muscular atrophy (SMA)

Learning and challenges	Implementation action	Process for sustainability in health practice and policy
The condition		
Clinical and neurophysiology assessments established that newborn infants with a genotype of 0 *SMN1* or two *SMN2* may have rapid disease progression and early clinical onset	Local NBS pathways reviewed to avoid delays (see below diagnosis and clinical care) Provision of information to families to identify symptoms of SMA while treatment plan being formulated	Pilot data informed application to add SMA to newborn bloodspot panel for national translation New knowledge corroborates NBS is the best tool for the early identification and treatment of SMA Development of national clinical guideline and pathway for routine care Dissemination and training of HCPs
Screening and diagnostic genetic testing		
Introduction of new assay to NBS was coupled with quality improvement activities Absence of established diagnostic pathway for rapid *SMN2* testing Absence of established diagnostic pathway for false‐positives Ensuring diagnostic testing is completed as soon as possible after referral to clinicians	Clinical and laboratory processes mapped end to end Incident monitoring, root cause analysis of the two individuals presenting with clinical symptoms identified underlying systematic weaknesses and random events, for which corrective actions and service improvements were identified. For example, DNA assays were integrated with a tandem mass spectrometry computerized tracking system within the NBS laboratory Sharing of molecular pathology and genetics expertise to investigate *SMN* locus in unusual cases Establish local *SMN2* copy number testing to expediate diagnostic pathways Increase flexibility and commitment from clinical and laboratory staff to deal with single urgent cases at short notice and change work patterns: clinical context and impact presented to laboratory; triage of *SMN1* diagnostic testing by NBS *SMN2* copy number: laboratory overtime shifts to initiate and run testing for two *SMN2* copies, urgent in‐hours testing for three or more *SMN2* copies Strong links between clinical and laboratory team with electronic and verbal communication for urgent testing and tracking progress	Standard operative procedure established National Association of Testing Authorities Australia accreditation for *SMN2* diagnostic reporting using QX200 Auto DG digital droplet PCR system within NBS laboratory. Consequently, diagnostic confirmation was contingent on corroboration of *SMN1* deletion in a different blood sample using assays with different *SMN1* primers Dissemination of diagnostic test protocol, including processes for *SMN1* and *SMN2* assessment and management of false‐positives NBS scientists familiar with tandem mass spectrometry and molecular genetic assays were trained to use the SMA and immunodeficiency assays within the NBS programme Maintain strong partnerships across clinical and laboratory health services to deliver high quality NBS care
SMA clinical care and supporting families as a central focus		
NBS identified families in remote areas, culturally and linguistically diverse backgrounds, infants born preterm, infants with serious concurrent illnesses Clinical uncertainty of more than three *SMN2* copies and management Limited availability of presymptomatic disease‐modifying therapy for three or more *SMN2* copies	Clinical team aware of individual circumstances to provide family‐centred care, including psychosocial support Clinical care included within current multidisciplinary neuromuscular service Increased flexibility of team to deal with urgent cases at short notice and change work patterns Clinical trials and compassionate access programmes supported access to disease‐modifying therapies Collaboration with policymakers to facilitate access to therapies	Dedicated team, communication strategy, and standard operating procedure to coordinate urgent and personalized clinic appointment The programme enables equity of diagnosis and access to health care Provision of family‐centred care Regulatory approval and reimbursement increased access to disease‐modifying therapies Development of national clinical guidelines and pathway for routine care
Impact on the programme as a whole		
SMA NBS achieved within theprogramme’s current consent and screening pathway No potential negative impacts upon other elements of the programme identified Costs, equipment, and training ascertained Cost‐effectiveness of screening not known	Use of existing information systems to collect and maintain data for monitoring, evaluation, and review Pilot budget included reagents, equipment, personnel, and training	Ongoing national funding for reagents, personnel, and equipment Evidence for cost‐effectiveness ongoing

HCP, health care professional; PCR, polymerase chain reaction.

#### Knowledge of the condition

**Figure 1:**
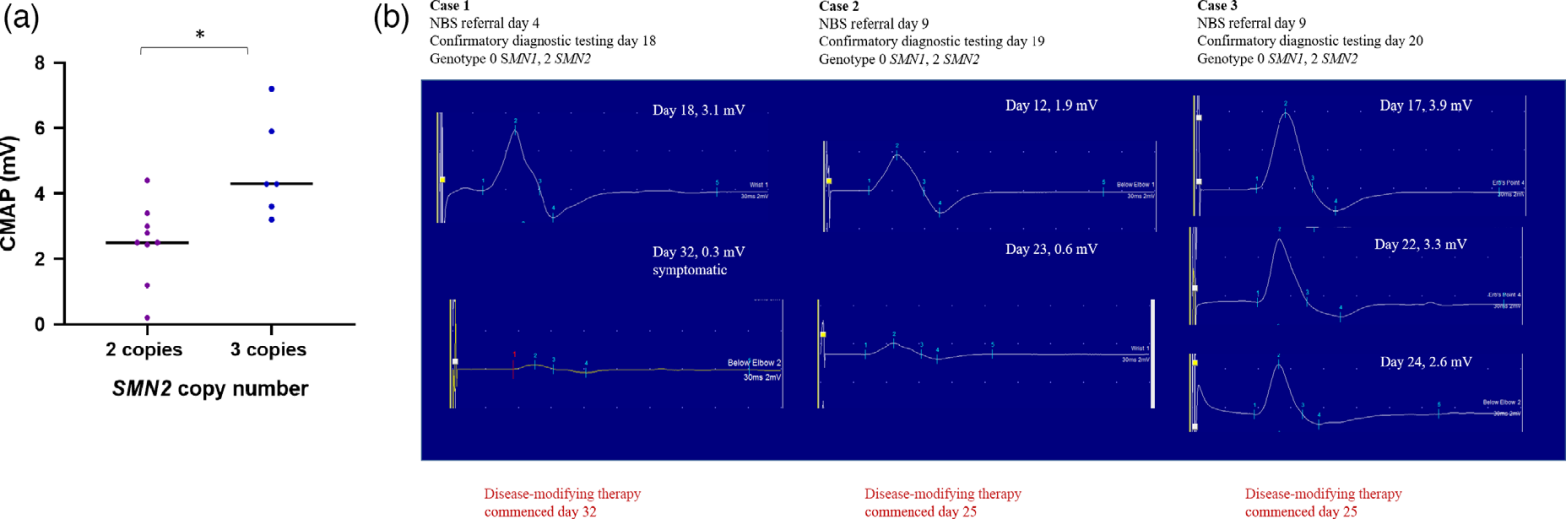
(a) Amplitudes of compound muscle action potentials (CMAPs) in newborn infants with two and three *SMN2* copy numbers. Dots, individual values; horizontal lines, mean values for the group. (b) Longitudinal ulnar nerve CMAPs in presymptomatic newborn infants predicted to have severe SMA (two *SMN2* copies). Ulnar CMAPs precipitously decreased in the second week and third week of life during initiation of disease‐modifying therapy, heralding symptom onset.

The incidence of SMA for those participating in the routine NBS programme was 1 in 11 458 (total 252 081, SMA 22, ascertained through NBS and subsequent clinical detection in this cohort). Among the 21 screen‐positive infants with homozygous *SMN1* exon 7 deletions, six showed clinical signs and symptoms of SMA disease onset (symptomatic) within the first 4 weeks of life. There were significant differences in CMAPs at the time of diagnosis, between infants with different SMA genotypes (two *SMN2* copies, mean tibialis anterior 2.5±1.2mV, range 0.2–4.4; three *SMN2* copies, mean tibialis anterior 4.3±1.5mV, range 3.2–7.2; *p*=0.02, Fig. 1a). Longitudinal assessments of ulnar CMAPs in three presymptomatic infants with two *SMN2* copies, concomitant with initiation of treatment over 7 to 14 days, demonstrated rapid and substantial reductions in amplitude (mean baseline 2.9±1.0mV, mean follow‐up 1.2±1.2mV; *p*=0.07), associated with symptom onset in one neonate (Fig. 1b). Our assessments of infants with SMA identified that the latent period may be short‐lived, emphasizing that NBS is necessary for early diagnosis, before or during latent motor neuron degeneration.

#### Screening and diagnostic testing for SMA

NBS covered more than 99.9% of all newborn infants in New South Wales. The NBS pilot protocol on all tested dried bloodspot cards had a sensitivity of 100%, specificity greater than 99.9%, false‐positive rate less than 0.001%, and positive predictive value of 95.5% (Table S3, online supporting information).

Rapid follow‐up quantification of *SMN2* copy number in all cases detected by NBS with homozygous *SMN1* exon 7 deletions is required for planning treatment, determining clinical subtypes, and follow‐up arrangements. A standard pathway for rapid diagnostic *SMN2* testing was not present before NBS, with accreditation for diagnostic testing held by one laboratory nationally. The median time to completing diagnostic workup (including *SMN2* copy number diagnostic results) was adversely influenced by interstate routine shipments occurring from Monday to Thursday before midday, which reduced operations during the COVID‐19 pandemic. Therefore, to enable efficiencies and additional testing capacity, the NBS laboratory sought and obtained National Association of Testing Authorities Australia accreditation for *SMN2* testing with digital droplet polymerase chain reaction on dried bloodspots. Dissemination of clinical findings led to increased flexibility and commitment from clinical and laboratory staff to deal with single urgent cases at short notice and change work patterns. The action was to triage *SMN1* diagnostic testing by *SMN2* copy number, such that laboratory overtime shifts initiated and ran testing for two *SMN2* copies and urgent in‐hours testing for three or more *SMN2* copies.

#### SMA clinical care and supporting families as a central focus

The families of infants with SMA identified by NBS came from a broad range of sociodemographic backgrounds including culturally and linguistically diverse communities and regional areas. Some newborn infants were born preterm or had serious concurrent illnesses. A recognized barrier was tailoring information to fit a variety of needs among families. Further implementation challenges included management of urgent referrals and diagnostic evaluations for infants with an initial positive screen to avoid delays, with a focus on family‐centred care.

**Figure 2:**
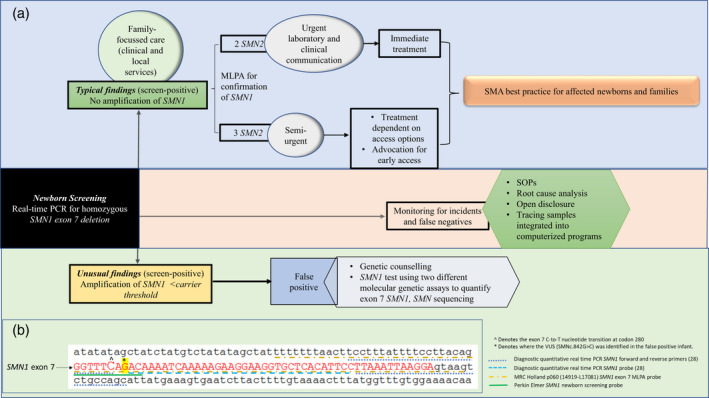
(a) Integration of the newborn bloodspot screening (NBS) spinal muscular atrophy (SMA) pilot programme into established NBS pathways. Assessment of *SMN2* copy number was determined on specimens of dried bloodspots with no functional copies of *SMN1* using digital droplet polymerase chain reaction (PCR). Diagnostic testing for National Association of Testing Authority‐accredited confirmation of genetic status involved corroboration of *SMN1* deletion results from whole blood samples (using a P060‐B2 SMA Multiplex Ligation Dependant Probe Amplification kit MRC‐Holland) by the NSW Health Pathology laboratory. *SMN2* copy number was diagnostically confirmed by quantitative PCR by the Victorian Clinical Genetics Service. After positive screening from dried bloodspots and diagnostic confirmation, families were provided detailed information about SMA to facilitate discussion of management options. (b) Sequence of *SMN1* exon 7 (capitalized) with flanking intronic regions showing location of probe/primers for relevant molecular genetic testing. The location of the single nucleotide variant (SMNc.842G>C) resulting in the NBS false‐positive is highlighted. The critical difference between *SMN1* and *SMN2* is C>T at nucleotide position 840 which alters splicing. The NBS primary assay used a PerkinElmer Eonis DNA extraction kit 3240‐0010 and an Eonis SCID‐SMA kit 3234‐0010 for quantitative real‐time PCR. MLPA, multiplex‐ligation dependent probe amplification. SOP, standard operating procedures.

Strong partnerships were created between NBS staff and neuromuscular specialists, successfully facilitated by integration of the NBS SMA pilot programme into established NBS pathways and neuromuscular models of care (Fig. 2). Before contacting families, close liaison between NBS services, local health care professionals (HCPs), and neuromuscular specialists identified the most appropriate clinical setting for an expeditious consultation for each family. Options included immediate referral to the neuromuscular team or, for those with difficulties travelling long distances, with the local paediatrician and specialist telehealth support. Increased flexibility of the team to deal with urgent and sometimes multiple cases at short notice and change work patterns ensured timely follow‐up despite logistical challenges, significant time commitments, unpredictability, and changes to health practices during the COVID‐19 pandemic.

The paediatric neuromuscular model of care included capability in clinical genetics to lead the investigation of unusual results, provide genetic counselling, and facilitate family cascade testing, supported by dedicated psychosocial input for families at a time of significant psychosocial stress. Our clinical therapeutic pathway and counselling was necessarily reliant on *SMN2* copy number in mostly presymptomatic infants, which introduced uncertainties in conversations with parents about clinical severity and long‐term outcomes.

Continuing care included nutritional, respiratory, and rehabilitative care, across a tertiary multidisciplinary team alongside collaboration and communication with community health care providers. Parents often sought guidance and reassurance from the neuromuscular team about parenting skills and common paediatric issues. The neuromuscular team provided clinical and emotional support and reassurance, alongside fostering engagement with relevant community early childhood HCPs.

Rapid developments in SMA treatment evolved during the pilot study, with overseas regulatory approval of gene therapy (onasemnogene abeparvovec) and risdiplam. Access to presymptomatic disease‐modifying therapies in Australia were through clinical trials or compassionate/managed access programmes before reimbursement of nusinersen in December 2020 (no more than *SMN2* copies). It continues to be important that access to approved and reimbursed therapy is proficient in condensing timelines and that logistical complexities in initiating disease‐modifying therapy for eligible patients are optimized. Ongoing collaboration between clinicians, families, industry, and policymakers continues to progress approval and reimbursement of SMA disease‐modifying therapies, aligned with evidence of efficacy, safety, and economic impact. We identified the need to develop a decision support analysis and national clinical guideline for SMA treatment and clinical care.

#### Impact of SMA screening on the NBS programme as a whole

Our pilot programme used current NBS consent and pathways and confirmed that these were suitable for the addition of SMA. The pilot programme did not affect the performance of the wider NBS programme, in particular the programme’s high participation rate sustained at more than 99.9%. The additional costs of screening were incorporated in the pilot programme budget, including purchase of reagents, new equipment, personnel, and training.

Across the four key domains, learnings were disseminated in real time with presentations at local, national, and international scientific meetings and webinars, with stakeholder meetings (between clinicians, advocacy organizations, and policymakers) providing an opportunity for feedback and further refinement of our processes. Our outcomes informed the application to the National Standing Committee of Screening to add SMA to the newborn bloodspot panel, together with a detailed economic evaluation.^15^ In May 2021 the Commonwealth Department of Health made a positive recommendation for the addition of SMA to routine NBS programmes by all Australian states.

## DISCUSSION

Therapeutic advances have rapidly transformed the landscape for SMA, prompting the development and initiation of NBS programmes for SMA globally.^2^ This study used an effectiveness‐implementation strategy to examine the clinical effectiveness of the NBS SMA programme and to collect data on its feasibility within the Australian health care system, focusing on the provision of consistent, equitable, and family‐centred care. Our study showed that the incidence of SMA was similar to panethnic estimates ascertained from retrospective studies of symptomatic individuals. Concerns about the potential of NBS programmes to overdiagnose, particularly in identifying individuals who would otherwise remain asymptomatic, seem to be unfounded.^16^ The incidence of newborn infants with more than three copies of *SMN2* was low in our population. The incidence of four *SMN2* copies is variable among NBS programmes.^17^, ^18^ Previous studies have identified differences in methodology accounting for discrepancies in individuals with higher copy numbers of *SMN2*; therefore, establishing standardization for *SMN2* copy number quantification is important for best practice. The population coverage for our study was high, in keeping with historically high rates of NBS uptake in Australia.^17^, ^19^

This study provided real‐time feedback to enhance NBS and clinical infrastructures that allow expedited access to screening, diagnostic, and therapeutic services. Critical insights into the brief latent period for those predicted to develop SMA type 1 were elucidated, highlighting the importance of urgent diagnostic testing and initiation of therapy, especially as irreversible motor neuron degeneration limits clinical benefit. CMAPs proved to be a useful adjunct to clinical characterization in identifying disease onset.

This study demonstrated that an NBS programme for SMA within the Australian health care system overcomes barriers of sociodemographic inequity. By using an implementation‐effectiveness model, we devised a pathway that integrated a biopsychosocial approach into current practice. This methodology provided an opportunity to transform health outcomes by delivering treatments targeted to the needs of individual patients on the basis of genetic, phenotypic, and psychosocial characteristics.^20^, ^21^

Several research priorities were recognized on the basis of challenges in clinical care. The need for initiatives to identify novel predictive and prognostic biomarkers to establish the impact of treatments alongside NBS on mortality and morbidity in SMA was identified. We used current information and evidence about *SMN2* copy number to predict phenotype and treatment, and acknowledged ambiguity for individual correlation in shared decision‐making.^22^ Additional biomarkers may be particularly valuable for individuals with more than three *SMN2* copies since these cohorts have the potential for considerable phenotypic heterogeneity. The feasibility and clinical utility of emerging biomarkers such as plasma neurofilament or rare genetic variants (e.g. c.859G>C and c.835‐44A>G) within an NBS programme thus requires investigation. Forthcoming clinical and economic evidence detailing the advantages of NBS coupled with treatment will be powerful engagement tools for routine adoption and sustainability. In addition, evidence‐based decision support guidelines and provision of contemporary written and electronic information for families,^23^ appropriate to national regulatory and reimbursement processes, are needed within a rapidly changing therapeutic landscape.

A multidisciplinary team, strong partnerships among stakeholders, continual robust lines of communication, and information exchange activities across clinical and laboratory staff were key to implementation. An important component of the Australian National Policy Framework for NBS is programme governance and organization, to ensure safety and quality.^24^ Our study emphasized the necessity of having robust laboratory and clinical processes to understand atypical screening results and clinical presentations of SMA.

NBS maximized opportunities for early intervention through timely and equitable SMA diagnosis, mitigating existing health inequities due to financial, geographical, cultural, and linguistic barriers to health care. Overcoming barriers of inequity is particularly important in the sphere of neurodisability where psychosocial disadvantage in addition to chronic disease culminates in reduced access to appropriate clinical care and social services.^25^ The success of SMA therapeutic intervention was reliant on best practice, facilitated by integration of NBS into contemporary multidisciplinary neuromuscular services to provide prompt and family‐centred care. For children and families with SMA this included capability in genetics, advanced therapeutics, and nutritional, respiratory, rehabilitative, and psychosocial care.^26^, ^27^

Our findings have health policy implications. The pilot NBS programme for SMA has created a standardized path to facilitate early diagnosis, targeted management of SMA, and translation for national routine adoption into clinical practice and health policy. This would benefit around 30 Australian families each year. An upcoming implementation challenge, intensified by the economic impact of the COVID‐19 pandemic, may be in obtaining prompt state‐based funding to maintain equity and sustainability.

Ongoing assessment of this programme will be necessary as further evidence, drug reimbursement guidelines, and clinical consensus statements emerge. Ultimately, strategic planning as well as multinational collaborations will continue to be critical to sustainable NBS across diverse health systems. Future research should also look at the impact of NBS SMA on long‐term prognosis for children diagnosed through NBS. The results from this study can be used as a blueprint for NBS programmes globally, as they work to expand the number of disorders screened, alongside providing a personalized model of care for identified individuals.

## Supporting information


**Table S1:** The decision criteria for the Australian National policy framework for newborn bloodspot screening

**Table S2:** Demographic and clinical characteristics of newborns screening positive and with diagnostic confirmation of spinal muscular atrophy

**Table S3:** Calculations of SMA screening test performance for the dried blood spot samples analysed for *SMN1* deletion

**Appendix S1:** Case study of an infant with an atypical false‐positive screening test for *SMN1* deletion.

## Data Availability

The data that support the findings of this study are available on request from the corresponding author. The data are not publicly available due to privacy or ethical restrictions.

## References

[1] Wilcken B , Wiley V . Newborn screening. Pathology. 2008;40(2):104–15. 18203033 10.1080/00313020701813743

[2] Farrar MA , Kiernan MC . Spinal muscular atrophy – the dawning of a new era. Nat Rev Neurol. 2020;16(11):593–4. 32978515 10.1038/s41582-020-00410-7

[3] De Vivo DC , Bertini E , Swoboda KJ , Hwu W‐L , Crawford TO , Finkel RS , et al. Nusinersen initiated in infants during the presymptomatic stage of spinal muscular atrophy: interim efficacy and safety results from the Phase 2 NURTURE study. Neuromuscul Disord. 2019;29(11):842–56. 31704158 10.1016/j.nmd.2019.09.007 PMC7127286

[4] Boemer F , Caberg J‐H , Dideberg V , Dardenne D , Bours V , Hiligsmann M , et al. Newborn screening for SMA in Southern Belgium. Neuromuscul Disord. 2019;29(5):343–9. 31030938 10.1016/j.nmd.2019.02.003

[5] Vill K , Kölbel H , Schwartz O , Blaschek A , Olgemöller B , Harms E , et al. One year of newborn screening for SMA – results of a German pilot project. J Neuromuscul Dis. 2019;6(4):503–15. 31594245 10.3233/JND-190428 PMC6918901

[6] Chien Y‐H , Chiang S‐C , Weng W‐C , Lee N‐C , Lin C‐J , Hsieh W‐S , et al. Presymptomatic diagnosis of spinal muscular atrophy through newborn screening. J Pediatr. 2017;190:124–9.e1. 28711173 10.1016/j.jpeds.2017.06.042

[7] Dangouloff T , Vrščaj E , Servais L , Osredkar D . Newborn screening programs for spinal muscular atrophy worldwide: where we stand and where to go. Neuromuscul Disord. 2021;31:574–82. 33985857 10.1016/j.nmd.2021.03.007

[8] Kellar‐Guenther Y , McKasson S , Hale K , Singh S , Sontag MK , Ojodu J . Implementing statewide newborn screening for new disorders: U.S. program experiences. Int J Neonatal Screen. 2020;6(2):35. 33073030 10.3390/ijns6020035 PMC7422992

[9] Andermann A , Blancquaert I , Beauchamp S , Déry V . Revisiting Wilson and Jungner in the genomic age: a review of screening criteria over the past 40 years. Bull World Health Organ. 2008;86:241–320. 10.2471/BLT.07.050112 PMC2647421 18438522

[10] Kariyawasam DST , Russell JS , Wiley V , Alexander IE , Farrar MA . The implementation of newborn screening for spinal muscular atrophy: the Australian experience. Genet Med. 2020;22(3):557–65. 31607747 10.1038/s41436-019-0673-0

[11] Curran GM , Bauer M , Mittman B , Pyne JM , Stetler C . Effectiveness‐implementation hybrid designs: combining elements of clinical effectiveness and implementation research to enhance public health impact. Med Care. 2012;50(3):217–26. 22310560 10.1097/MLR.0b013e3182408812 PMC3731143

[12] Ogrinc G , Davies L , Goodman D , Batalden P , Davidoff F , Stevens D . SQUIRE 2.0 (Standards for QUality Improvement Reporting Excellence): revised publication guidelines from a detailed consensus process. BMJ Qual Saf. 2016;25(12):986–92. 10.1136/bmjqs-2015-004411 PMC5256233 26369893

[13] Graham ID , Logan J , Harrison MB , Straus SE , Tetroe J , Caswell W , et al. Lost in knowledge translation: time for a map? J Contin Educ Health Prof. 2006;26(1):13–24. 16557505 10.1002/chp.47

[14] Newborn Bloodspot Screening National Policy Framework [Internet]; 2018. https://www.health.gov.au/resources/publications/newborn‐bloodspot‐screening‐national‐policy‐framework. Accessed 17th Dec 2020.

[15] Shih STF , Farrar MA , Wiley V , Chambers G . Newborn screening for spinal muscular atrophy with disease‐modifying therapies: a cost‐effectiveness analysis. J Neurol Neurosurg Psychiatry. 2021;92(12):1296–304. 34321343 10.1136/jnnp-2021-326344

[16] Sugarman EA , Nagan N , Zhu H , Akmaev VR , Zhou Z , Rohlfs EM , et al. Pan‐ethnic carrier screening and prenatal diagnosis for spinal muscular atrophy: clinical laboratory analysis of >72,400 specimens. Eur J Hum Genet. 2012;20(1):27–32. 21811307 10.1038/ejhg.2011.134 PMC3234503

[17] Boemer F , Caberg J‐H , Dideberg V , Dardenne D , Bours V , Hiligsmann M , et al. Newborn screening for SMA in Southern Belgium. Neuromuscul Dis. 2019;29(5):343–9. 10.1016/j.nmd.2019.02.003 31030938

[18] Vill K , Kölbel H , Schwartz O , Blaschek A , Olgemöller B , Harms E , et al. One year of newborn screening for SMA – results of a German pilot project. J Neuromuscul Dis. 2019;6(4):503–15. 31594245 10.3233/JND-190428 PMC6918901

[19] Vill K , Schwartz O , Blaschek A , Gläser D , Nennstiel U , Wirth B , et al. Newborn screening for spinal muscular atrophy in Germany: clinical results after 2 years. Orphanet J Rare Dis. 2021;16(1):153. 33789695 10.1186/s13023-021-01783-8 PMC8011100

[20] Bronfenbrenner U . Ecology of the family as a context for human development: research perspectives. Dev Psychol. 1986;22:723–42.

[21] Jameson JL , Longo DL . Precision medicine–personalized, problematic, and promising. N Engl J Med. 2015;372(23):2229–34. 26014593 10.1056/NEJMsb1503104

[22] Cusco I , Bernal S , Blasco‐Perez L , Calucho M , Alias L , Fuentes‐Prior P , et al. Practical guidelines to manage discordant situations of SMN2 copy number in patients with spinal muscular atrophy. Neurol Genet. 2020;6(6):e530. 33324756 10.1212/NXG.0000000000000530 PMC7713720

[23] Kariyawasam DS , D’Silva AM , Vetsch J , Wakefield CE , Wiley V , Farrar MA . “We needed this”: perspectives of parents and healthcare professionals involved in a pilot newborn screening program for spinal muscular atrophy. EClinicalMedicine. 2020;33:100742. 10.1016/j.eclinm.2021.100742 PMC8020144 33842861

[24] Department of Health. Australian Health Ministers Advisory Council . Newborn bloodspot screening national policy framework; 2018. p. 1–73.

[25] Turner J , Kelly B . Emotional dimensions of chronic disease. West J Med. 2000;172(2):124–8. 10693376 10.1136/ewjm.172.2.124 PMC1070773

[26] Finkel RS , Mercuri E , Meyer OH , Simonds AK , Schroth MK , Graham RJ , et al. Diagnosis and management of spinal muscular atrophy: Part 2: pulmonary and acute care; medications, supplements and immunizations; other organ systems; and ethics. Neuromuscul Disord. 2018;28(3):197–207. 29305137 10.1016/j.nmd.2017.11.004

[27] Mercuri E , Finkel RS , Muntoni F , Wirth B , Montes J , Main M , et al. Diagnosis and management of spinal muscular atrophy: Part 1: recommendations for diagnosis, rehabilitation, orthopedic and nutritional care. Neuromuscul Disord. 2018;28(2):103–15. 29290580 10.1016/j.nmd.2017.11.005

[28] Smith M , Calabro V , Chong B , Gardiner N , Cowie S , du Sart D . Population screening and cascade testing for carriers of SMA. Eur J Hum Genet. 2007;15(7):759–66. 17392705 10.1038/sj.ejhg.5201821

[29] Kraszewski JN , Kay DM , Stevens CF , Koval C , Haser B , Ortiz V , et al. Pilot study of population‐based newborn screening for spinal muscular atrophy in New York state. Genet Med. 2018;20(6):608–13. 29758563 10.1038/gim.2017.152

