# Supplementary material for: Integrating newborn screening for spinal muscular atrophy into health care systems: an Australian pilot programme
Source: Dev Med Child Neurol. 2021 Nov 28;64(5):625–32. doi: 10.1111/dmcn.15117 (PMC9299803; doi:10.1111/dmcn.15117)
Supplement: Supplementary file 1 — Table S1: The decision criteria for the Australian National policy framework for newborn bloodspot screening [file DMCN-64-625-s001.pdf]

Supplementary table 1: **The decision criteria for the Australian National policy framework for newborn bloodspot screening (24).**

Fundamental principles take into account the condition, the screening test, the intervention, and the benefits of screening for SMA weighed against its impact on the whole NBS program (9).

**Decision making criteria**	**Principles**
The condition	The condition should be a serious health problem that leads to significant morbidity or mortality.<br>There should be a benefit to conducting screening in the newborn period.<br>The natural history of the condition, including development from latent to declared disease, should be adequately understood.
The screening test	There should be a suitable test protocol to identify the presence of the condition.<br>The test protocol should, on balance, be socially and ethically acceptable to health professionals and the public.
The intervention	Health care services for diagnosis and management should be available so that these services can be offered if there is an abnormal screening result.<br>There should be an accepted intervention for those diagnosed with the condition.
The impact on the whole program	The benefit of screening a condition must be weighed against its impact on the program.<br>What other information relevant to decision making should be considered that has not been captured elsewhere?
