# Supplementary material for: Integrating newborn screening for spinal muscular atrophy into health care systems: an Australian pilot programme
Source: Dev Med Child Neurol. 2021 Nov 28;64(5):625–32. doi: 10.1111/dmcn.15117 (PMC9299803; doi:10.1111/dmcn.15117)
Supplement: Supplementary file 2 — Table S2: Demographic and clinical characteristics of newborns screening positive and with diagnostic confirmation of spinal muscular atrophy [file DMCN-64-625-s003.pdf]

Supplementary table 2: **Demographic and clinical characteristics of newborns screening positive and with diagnostic confirmation of spinal muscular atrophy.**

*4*SMN2*: This infant was screened with 3 copies of *SMN2* on qPCR (on DBS). Diagnostic confirmation by ddPCR revealed 4 copies of *SMN2*

** Age at diagnostic confirmation is equivalent to time from birth to obtaining *SMN1* diagnostic result.

	**Participants**
	n = 21
**Sex**	
Female	10 (48 %)
Male	11 (52 %)
***SMN2* copy number**	
2	12 (57.2 %)
3	8 (38 %)
*4	1 (4.8%)
**Age at screening result**	3 (2-15), 3.28
****Age at diagnostic confirmation** (days)<br>Median, (range), SD	15 (10-23), 3.84
**Age at therapeutic intervention** (days)<br>Median, (range), SD	25 (15-39), 7.46
