# Supplementary material for: Integrating newborn screening for spinal muscular atrophy into health care systems: an Australian pilot programme
Source: Dev Med Child Neurol. 2021 Nov 28;64(5):625–32. doi: 10.1111/dmcn.15117 (PMC9299803; doi:10.1111/dmcn.15117)
Supplement: Supplementary file 3 — Table S3: Calculations of SMA screening test performance for the dried blood spot samples analysed for SMN1 deletion [file DMCN-64-625-s004.pdf]

Supplementary table 3: Calculations of SMA screening test performance for the dried blood spot samples analysed for *SMN1* deletion *

	Newborn screen positive	Newborn screen negative	
Confirmed SMA	21	0	Sensitivity = 100%
No SMA	1	252058	Specificity >99.9%
	Precision (Positive predictive value) = 95.5 %	Negative Predictive value = 100%	Accuracy >99.9%

* Total number of NBS samples analysed = 252080. The NBS sample not analysed for *SMN1* during establishment of pilot and before integration into lab computerised systems is not included.
