# Supplementary material for: Integrating newborn screening for spinal muscular atrophy into health care systems: an Australian pilot programme
Source: Dev Med Child Neurol. 2021 Nov 28;64(5):625–32. doi: 10.1111/dmcn.15117 (PMC9299803; doi:10.1111/dmcn.15117)
Supplement: Supplementary file 4 — Appendix S1: Case study of an infant with an atypical false‐positive screening test for SMN1 deletion. [file DMCN-64-625-s002.pdf]

Box 1: Case study of an infant with an atypical false positive screening test for *SMN1* deletion

Neonate, born at 39 weeks' gestation to consanguineous parents with no family history of SMA.

First tier SMN1 screening

- NBS for SMA with a quantitative PCR indicated an unusual signature for exon 7 *SMN1* deletion, verified on a repeat DBS.
- The amplification curve was below that seen in SMA carriers with a heterozygous exon 7 *SMN1* deletion, and not typical for homozygous exon 7 *SMN1* deletions.
- Equivocal screening results prompted discussions with neuromuscular experts, geneticists and the NBS laboratory.
- Parents were requested to attend the neuromuscular clinic for clinical assessment of their newborn and themselves and diagnostic determination of *SMN1* gene copy number

Diagnostic SMN1 testing

- Diagnostic testing for *SMN1* deletion reported the patient was a heterozygous carrier of *SMN1* exon 7 deletion (MRC Holland P060-B2 SMA Multiplex Ligation-dependent Probe Amplification (MLPA) kit). However, testing in the parents was non conclusive (not clearly normal nor consistent with heterozygous carrier status).
- A second diagnostic laboratory repeated *SMN1* testing using a different methodology (quantitative real time PCR) (28), and this showed that the infant had 2 copies of *SMN1* exon 7, with no evidence of *SMN1* deletion. Parental testing also showed that they both have 2 copies of *SMN1*, not suggestive of being a carrier of an *SMN1* deletion.
- The parental consanguinity raised the possibility of the infant being homozygous for a rare *SMN1* gene sequence variant resulting in only partial binding, located under the binding sites of a PCR probe/primer from the NBS quantitative PCR assay and another from the MLPA assay, but which was not within the probe/primer site locations for the diagnostic quantitative PCR assay.
- Sanger sequencing of SMN was then arranged to examine this possibility.

Sanger sequencing of* SMN *gene

- A variant c.842G>C was identified which may be present in either the *SMN1* or *SMN2* gene, as the assay used is unable to distinguish between *SMN1* and *SMN2* genes. The infant and parental allelic ratios at c.842 were ~1:1 and 3:1, respectively.
- The variant was present in the infant and both parents and based on the ratio of *SMN1* to *SMN2* and sequencing patterns, it was concluded that the variant was homozygous in the infant, and heterozygous in each parent.

Annotation of the c.842G>C variant and classification as a variant of uncertain significance (VUS) assuming it is in SMN1

- A missense variant, NM_000344.3(*SMN1*):c.842G>C was identified in the *SMN1* gene (GRCh37 chr:70247775)
- This is predicted to result in an amino acid change from arginine to threonine at position 281; NP_000335.1 (*SMN1*): p (Arg281Thr).
- In silico software predictions of the pathogenicity of this variant were conflicting. This variant is not located in an established domain, motif, hotspot or informative constraint region.
- This variant has been previously reported in an unaffected individual in a population based NBS program and described as a VUS (29). It has not been reported in clinically affected SMA patients.
- No published functional evidence has been identified for this variant.
- Based on current information, the variant was classified as a VUS in the *SMN1* gene.

Summary of all test results explaining NBS false positive

- This SMA NBS result represents a false positive NBS result as the infant does not have homozygous deletion of *SMN1*.
- The atypical false positive NBS result (and inconclusive MLPA result) is explained by the single nucleotide variant within the region of the MLPA probe and Perkin Elmer NBS probe, which is not located within the region of the primers or probe of the quantitative real time PCR used by the second diagnostic laboratory (28) – hence their results showing infant and parents both having 2 copies of *SMN1*.

Clinical summary

- Clinical and neurophysiological examinations of the infant in the first 12 months were normal, with the child starting to walk, and a diagnosis of SMA types 1 and 2 were ruled out.
- The possibility of the infant developing later-onset SMA (SMA type 3 and 4) could not be excluded, and neuromuscular surveillance suggested.
- Prenatal diagnosis was not offered for a subsequent pregnancy.
